# Supplementary material for: Reducing non-communicable diseases among Palestinian populations in Gaza: A participatory comparative and cost-effectiveness modeling assessment
Source: PLOS Glob Public Health. 2024 May 2;4(5):e0003168. doi: 10.1371/journal.pgph.0003168 (PMC11065248; doi:10.1371/journal.pgph.0003168)
Supplement: S1 Text — (DOCX) [file pgph.0003168.s002.docx]

**Supporting Information Text**

*Reviews of intervention effectiveness and cost data.*

We conducted a review of peer-reviewed literature to identify the impact of each simulated intervention, itemized below. We followed the PRISMA guidelines [[1]](https://www.zotero.org/google-docs/?Hn1Ozx) and searched PubMed.gov and Google Scholar, as well as using two artificial intelligence tools (elicit.org and consensus.app) for relevant articles using combinations of the search terms specified under each intervention subheading below. We isolated the subset of studies that indicated a low risk of bias using the Cochrane Risk of Bias tool for randomized controlled trials [[2]](https://www.zotero.org/google-docs/?XXz9vC), and the ROBINS-I tool for non-randomized studies [[3]](https://www.zotero.org/google-docs/?AdlzXn).

1. WHO Intervention 1.4 Eliminate exposure to second-hand tobacco smoke in all indoor workplaces, public places, and public transport.

We reviewed the impact of eliminating exposure to second-hand tobacco smoke in all indoor workplaces, public places, and public transport by finding relevant peer-reviewed articles using combinations of the search terms "second-hand smoke," "smoke-free policies," "indoor workplaces," "public places," "public transport," and "tobacco smoke exposure" alongside each of the NCDs. Eighty-two relevant results were found, which included seven international systematic reviews [[4–10]](https://www.zotero.org/google-docs/?SO6EB8). The subset of studies included in our model were cohort studies estimating a relative risk of 0.72 (95% CI: 0.56, 0.93) for CVD risk among men and 0.70 (0.54, 0.90) in CVD risk among women after smoke-free policies [[11]](https://www.zotero.org/google-docs/?AUYayo), alongside cohort studies suggesting smokefree legislation was also associated with a RR of 0.95 (0.91, 0.99) in the risk of asthma exacerbations [[12]](https://www.zotero.org/google-docs/?i6CGp9). Extensive prior reviews did not find a significant association between passive smoking or smoke-free legislation on the cancer outcomes studied in this assessment [[13]](https://www.zotero.org/google-docs/?Uth57H). For cost evaluation, we updated a prior WHO evaluation of implementation cost for smoke-free policies [[14]](https://www.zotero.org/google-docs/?13ju98), which had indicated a $0.13 per person annual cost for smoke-free legislation in 2005 in international dollars (0.05, 0.32), which in 2023 dollars amounts to $0.20 per person per year when adjusted for inflation and adjusted for the Palestinian GDP purchasing power parity (0.08, 0.49). The cost estimate included costs of administration, monitoring and evaluation, supervision and training, with the uncertainty range primarily driven by variations in salary unit costs for civil service workers instituting the policy, as reported previously by the WHO CHOICE 2020 data and methods update [[15]](https://www.zotero.org/google-docs/?lOIDUx).

1. WHO Intervention 2.11 Implement nutrition education and counseling in different settings to increase the intake of fruits and vegetables

To identify the impact of implementing nutrition education and counseling in different settings, such as preschools, schools, workplaces, and hospitals, we used combinations of the search terms "nutrition education," "nutrition counseling," "preschools," "schools," "workplaces," "hospitals," "fruit intake," and "vegetable intake" alongside each of the NCDs We identified fifty-four relevant articles, including seven international systematic reviews [[16–22]](https://www.zotero.org/google-docs/?2rUrbK) and four relevant randomized trials [[20,23–25]](https://www.zotero.org/google-docs/?9AiWQa). We simulated the intervention impact in Gaza by first estimating the increase in the proportion of the population who consume fruits and vegetables daily, then estimating the impact of daily consumption on the simulated NCD outcomes. For the intervention impact, we utilized a controlled study suggesting a typical 4.6% increase in the proportion of the population with daily fruit and vegetable intake (95% CI: 1.6%, 7.6%), among persons in the WHO EMRO region when subjected to a standardized WHO-guideline-based nutritional education and counseling intervention in workplace settings [[26]](https://www.zotero.org/google-docs/?fuy2a9). For each person with daily intake, the associated relative risk from prospective cohort studies was 0.82 (0.76, 0.89) for CVD [[27]](https://www.zotero.org/google-docs/?jjrZ8M), 0.86 for diabetes (0.77, 0.97) [[28]](https://www.zotero.org/google-docs/?khnLCJ), 0.89 for incident breast cancer (0.83, 0.96) [[29]](https://www.zotero.org/google-docs/?6bjdwF), and 0.91 for incident colorectal cancer (0.85, 0.97) [[30]](https://www.zotero.org/google-docs/?vsX8SV). We did not observe evidence for subsequent changes in mortality risk ratio for any of the studied NCDs within the reviews. For cost evaluation, we updated a prior evaluation of implementation cost for nutrition education and counseling in workplaces and adjusted the estimate of 57 Euros per person per year in the setting to the Palestine GDP purchasing power parity and for inflation to an estimate $4 per person per year in Gaza in 2023 [[31]](https://www.zotero.org/google-docs/?Ksk0NH). The estimate included costs incurred in two main phases: set-up and maintenance. Within these phases, five cost categories were considered. Nutritionist costs accounted for staff costs, including time spent on food product and menu analysis, staff training, individual consultations, group presentations, and monitoring adherence. Catering costs encompassed staff costs for menu changes, display of nutritional information, and training on portion control. Management stakeholder costs involved the time workplace staff spent in meetings to coordinate the intervention. Employee costs included the time costs associated with employees participating in the intervention during working hours, which could include attending nutrition consultations and group presentations. Lastly, printing and material costs were related to the procurement and printing of materials for displaying detailed nutritional information in the workplace.

1. WHO Intervention 2.13 Implement mass media campaign on healthy diets, including social marketing to reduce the intake of total fat, saturated fats, sugars and salt, and promote the intake of fruits and vegetables

To identify the impact of implementing mass media campaign on healthy diets, including social marketing to reduce the intake of total fat, saturated fats, sugars and salt, and promote the intake of fruits and vegetables, we used combinations of the search terms "mass media", "social marketing", "healthy diet", "fat intake", "sugar intake", "salt intake", and "fruit and vegetable intake" alongside each of the NCDs. We identified six international systematic reviews relevant to the question [[32–38]](https://www.zotero.org/google-docs/?IwjzOB). We used results from the systematic review and meta-analysis with most comprehensive coverage of relevant populations, suggesting the typical effect of mass media campaigns is to increase the portion of people with daily fruit and vegetable intake by 7% (95% CI: 4%, 9%) [[38]](https://www.zotero.org/google-docs/?C5lSxa). We then used the same downstream relative risk estimates on NCD outcomes as described for intervention 2.11, based on the proportion of the population with daily intake. For cost evaluation, we updated a prior WHO evaluation of implementation cost for mass media campaigns in the WHO EMR region, priced at $0.18 per person per year [[39]](https://www.zotero.org/google-docs/?LoqGIa), which we updated to $0.30 per person per year by adjusting for inflation and the GDP purchasing power parity in Palestine. The prior analysis included the standard ingredients approach developed by the WHO, which included estimates of common media planning and execution infrastructure (labor and office space), their unit costs, and the scaled quantities of effective media-related costs per person to achieve the effectiveness observed in the prior meta-analytic assessments. Program-level costs include administration, training, and media design and publication/dissemination in the form of both print and broadcast (radio, television, Internet) media types.

1. WHO Intervention 3.1 Implement community wide public education and awareness campaign for physical activity which includes a mass media campaign combined with other community based education, motivational and environmental programmes aimed at supporting behavioral change of physical activity levels

To identify the impact of implementing community wide public education and awareness campaigns for physical activity, we used combinations of the search terms "community-wide campaigns", "public education", "awareness campaigns", "physical activity promotion", "mass media campaigns", "community-based education", "motivational programs", "environmental programs", "behavioral change", "physical activity levels", "health promotion", "exercise promotion", "active living", "social support for physical activity", "community interventions", "health communication", "media and physical activity", "social marketing for physical activity", "walking groups", "exercise buddy system" alongside each of the NCDs. We identified five international systematic reviews relevant to the question [[40–44]](https://www.zotero.org/google-docs/?2WWAew). The most relevant review of randomized trials suggested that with the intervention, the median net increase in the percentage of people who reported being physically active per standards of international leisure time physical activity thresholds would be 4.2% (95% CI: 2.9% to 9.4%; at least 150 to 300 minutes of moderate-intensity aerobic physical activity, or at least 75 to 150 minutes of vigorous-intensity aerobic physical activity, or an equivalent combination of both, per week) [[44,45]](https://www.zotero.org/google-docs/?eombfw). Based on a meta-analyses, we estimated the RR of CVD among this group to be 0.76 (95% CI: 0.70–0.82) among men and 0.73 (0.68–0.78) among women [[46]](https://www.zotero.org/google-docs/?bEI9Ay); for diabetes to be 0.69 (0.58, 0.83) [[47]](https://www.zotero.org/google-docs/?5XbEWQ); for asthma/COPD exacerbation to be 0.72 (0.53, 0.97) [[48,49]](https://www.zotero.org/google-docs/?metM2Q); for breast cancer to be 0.81 (0.69, 0.96) [[50]](https://www.zotero.org/google-docs/?6Ikez0); and for colorectal cancer to be 0.76 (0.72, 0.81) [[51]](https://www.zotero.org/google-docs/?rTnwRw). Also per a systematic review, the estimated cost per person per week was $0.31 (0.07, 0.56) in 2020, which when updated to the Palestinian GDP per capita purchasing power parity and inflation was estimated to be $18.90 ($18.65, $19.15) per person per year. Costs included the mass media parameters described earlier among nutrition mass media campaigns, tailored to media promotions and community-wide interventions and promotions related to physical activity.

1. WHO intervention 3.2 Provide physical activity counseling and referral as part of routine primary health care services through the use of a brief intervention

To identify the impact of providing physical activity counseling and referral as part of routine primary health care services through the use of a brief intervention, we used combinations of the search terms "physical activity counseling", "referral", "primary care", and "brief intervention" alongside each of the NCDs. We identified five international systematic reviews relevant to the question [[52–56]](https://www.zotero.org/google-docs/?VVYYSo). The most relevant review of randomized trials suggested that with the intervention, the median net increase in the percentage of people who reported being physically active per standards of international leisure time physical activity thresholds would be 11% (95% CI: 4%, 17%) [[52]](https://www.zotero.org/google-docs/?vbNU9z). The resulting relative risk reductions for NCD outcomes were the same per person with increased physical activity as for WHO Intervention 3.1 specified above. Because Gaza lacks a referral outlet to provide the physical activity services, we included the estimated cost per person per month from not only screening and referral but also from delivering the service itself of physical activity counseling. The closest relevant cost estimation included an estimate of CA$91.43 per person per month in 2012, which when adjusted for GDP per capita purchasing power parity in Palestine and inflation amounted to $110 per person per year in Palestine ($100, $120) [[57]](https://www.zotero.org/google-docs/?4nt86o). The cost assessment included office space, equipment, operating costs including supervision, connectivity resources (telephone, Internet), physical activity counselor training including professional development, and labor costs. We subset the costs and DALY benefits to those reporting visiting primary care practices in the household survey.

1. WHO Intervention 6.2 Screening with mammography (once every 2 years for women aged 50-69 years) linked with timely diagnosis and treatment of breast cancer

To identify the impact of screening with mammography linked with timely diagnosis and treatment of breast cancer, we used combinations of the search terms “mammography”, “screening” and “breast cancer”. We identified four international systematic reviews relevant to the question [[58–61]](https://www.zotero.org/google-docs/?g352Yt) as well as several prospective cohort studies and randomized trials [[62–70]](https://www.zotero.org/google-docs/?mZOgW5). We included an inclusive meta-analysis-based summary estimate of the impact of every 2 year mammograms for age group 50-69 years old that estimated a breast cancer mortality rate ratio of 0.72 (95% CI: 0.64, 0.79) [[64]](https://www.zotero.org/google-docs/?sH93Uf). The closest relevant cost estimation included an estimate of $70 per person per year in Jordan in 2021, which when adjusted for GDP per capita purchasing power parity in Palestine and inflation amounted to $20 per person per screening year in Palestine (95% CI: $15, $25) [[71]](https://www.zotero.org/google-docs/?mG7liA). Costs and DALYs for breast cancer were limited to women, with mammography costs limited to those aged 50-69 years of age. Costs incorporated screening and counseling, technology and equipment expenditure, labor costs including specialty costs related to both administration of screening and interpretation, and subsequent implications for breast cancer management among incident cases as well as costs associated with false-positive results.

1. WHO Intervention 6.3 Treatment of colorectal cancer stages I and II with surgery +/- chemotherapy and radiotherapy

To identify the impact of treatment of colorectal cancer stages I and II with surgery +/- chemotherapy and radiotherapy, we used combinations of the search terms "colorectal cancer", "colon cancer", "rectal cancer", "stage I colorectal cancer", "stage II colorectal cancer", "treatment", "surgical resection", "resection", "colectomy", "hemicolectomy", "chemotherap*", "5-fluorouracil", "capecitabine", "oxaliplatin", "irinotecan", "radiotherap*", "radiation therap*", "adjuvant therap*", "neoadjuvant therap*", "survival", "prognosis", "recurrence", "effectiveness". We identified 11 international systematic reviews relevant to the question [[72–81]](https://www.zotero.org/google-docs/?J56uCJ). We utilized the overall statistical assessment of these reviews indicating that treatment of colorectal cancer stages I and II would be expected to improve the 5-year relative survival rate to 65% [[82]](https://www.zotero.org/google-docs/?o9ZEw7). The closest relevant cost estimation included four assessments from neighboring countries [[83–86]](https://www.zotero.org/google-docs/?0H5OWZ), which when adjusted for GDP per capita purchasing power parity in Palestine and inflation amounted to $12,262 per incident case of stage I or II colorectal cancer (including probability and cost of recurrence). Cost incorporated direct medical costs including personnel, treatment infrastructure, and treatment materials including medications and hospitalization costs; and indirect costs to patients including potentially lost employment, out of pocket expenditures, and indirect costs to the household.

1. WHO Intervention 6.4 Treatment of breast cancer stages I and II with surgery +/- systemic therapy

To identify the impact of treatment of breast cancer stages I and II with surgery +/- systemic therapy, we used combinations of the search terms "breast cancer", "early-stage breast cancer", "stage I breast cancer", "stage II breast cancer", "surgery for breast cancer", "systemic therapy for breast cancer", "neoadjuvant therapy for breast cancer", "adjuvant therapy for breast cancer", "breast-conserving surgery", "mastectomy for breast cancer", "lumpectomy for breast cancer", "sentinel lymph node biopsy", "axillary lymph node dissection", "drug therapy for breast cancer", "chemotherapy for breast cancer", "hormonal therapy for breast cancer", "targeted therapy for breast cancer", "radiation therapy for breast cancer", "treatment outcomes for breast cancer stages I and II", and "prognosis for early-stage breast cancer". We identified seven international systematic reviews relevant to the question [[87–93]](https://www.zotero.org/google-docs/?GFbfji). We utilized the overall statistical assessment of these reviews indicating that treatment of breast cancer stages I and II would be expected to improve the 5-year relative survival rate to 92% [[94]](https://www.zotero.org/google-docs/?A08cN4). The closest relevant cost estimate was from Saudi Arabia [[85]](https://www.zotero.org/google-docs/?63B1bz), which when adjusted for GDP per capita purchasing power parity in Palestine and inflation amounted to $1,423 per incident case of stage I or II breast cancer (including probability and cost of recurrence). Cost incorporated direct and indirect costs as with the colorectal cancer cost assessment.

1. WHO Intervention 7.3 Treatment of asthma using low dose inhaled beclometasone and short acting beta agonist

To identify the impact of treatment of asthma using low dose inhaled beclometasone and short acting beta agonist, we used combinations of the search terms "asthma", "low dose inhaled beclometasone", "short acting beta agonist", and "treatment". We identified four international systematic reviews and seven large randomized trials relevant to the question [[95–105]](https://www.zotero.org/google-docs/?9VGtlN). We used the most comprehensive and updated systematic review estimate that suggested a 15% reduction in the hazard ratio for a severe exacerbation with as-needed inhaled corticosteroid-formoterol [[105]](https://www.zotero.org/google-docs/?EjrEPQ). The closest relevant cost estimates were from Abu Dhabi [[106,107]](https://www.zotero.org/google-docs/?tYphEm), which when adjusted for GDP per capita purchasing power parity in Palestine and inflation amounted to $24 per person per year. Costs incorporated medication use; healthcare expenditures including hospitalization, emergency room visit and outpatient costs; and indirect costs from work absenteeism.

*Risk score calculation approaches*

1. CVD

We reviewed the performance of several CVD risk prediction models including the Framingham risk score [[108]](https://www.zotero.org/google-docs/?U7SzHe), WHO/ISH [[109]](https://www.zotero.org/google-docs/?rlcLXx), ACC/AHA ASCVD risk prediction model [[110]](https://www.zotero.org/google-docs/?CteIO1), and Globorisk [[111,112]](https://www.zotero.org/google-docs/?dBHYXr), and chose the Globorisk model due to its validation in Middle Eastern populations. We specifically utilized the laboratory-based Globorisk 10-year risk score, which is a CVD risk score calculator that predicts the risk of first myocardial infarction or stroke in 182 countries [[111,112]](https://www.zotero.org/google-docs/?gOXp1f). It uses information on a person's country of residence, age, sex, smoking, diabetes, blood pressure, and lipid profile to predict risk. We used multiple imputation with chained equations for those individuals without a lipid profile in our dataset. The Globorisk model was developed through a pooled analysis of prospective cohorts and health examination surveys [[111]](https://www.zotero.org/google-docs/?d7aDIh). It has been validated and calibrated using data from various countries and regions, including–geographically closest to Gaza–in Iran [[113]](https://www.zotero.org/google-docs/?zjX5YH). The Globorisk model is country-specific, and we utilized the R package `globorisk` to calculate the 10-year risk of CVD for individuals in these countries [[114]](https://www.zotero.org/google-docs/?AQEk2Y). We averaged from three country estimates–from Lebanon, Syria and Jordan–to estimate risk for individuals in Gaza.

1. Diabetes

We reviewed a series of diabetes risk prediction models including the AUSDRISK [[115]](https://www.zotero.org/google-docs/?iOLY1u), San Antonio [[116]](https://www.zotero.org/google-docs/?qIjLJH), ARIC [[117]](https://www.zotero.org/google-docs/?pexDsP), and FINDRISC models [[118]](https://www.zotero.org/google-docs/?5QHPU3), and chose a model validated among Middle Eastern populations. utilized the Finnish Diabetes Risk Score (FINDRISC), which is a questionnaire-based tool designed to identify individuals at high risk of developing type 2 diabetes (T2D) [[118]](https://www.zotero.org/google-docs/?Ks9zGD). FINDRISC incorporates the patient’s age, body mass index (BMI), waist circumference, physical activity (30 minutes or more daily), dietary consumption of fruits and vegetables (daily consumption of any amount, or not), use of antihypertensive medication (yes/no), history of high blood glucose (yes/no), and family history of diabetes (with higher scoring for first-degree relatives versus further degrees). Regarding validation in Middle Eastern populations, a study conducted in Lebanon found that FINDRISC was a valid screening tool for undiagnosed T2D and metabolic syndrome in a Lebanese community of working people [[119]](https://www.zotero.org/google-docs/?9FiIV7). Another study in Jordan assessed the usefulness of FINDRISC in a dental setting, but the results do not provide specific information on its validation in this population [[119]](https://www.zotero.org/google-docs/?hmjyH7). The tool has been more extensively validated and demonstrated good predictive value in detecting undiagnosed T2D and predicting the incidence of T2D in Mediterranean populations. For example, the area under the receiver operating characteristic (AUROC) curve was 0.72 for detecting undiagnosed T2D in Greece [[120]](https://www.zotero.org/google-docs/?4l4g2k) and 0.75 for predicting incident T2D in southern Spain [[121]](https://www.zotero.org/google-docs/?e1bCKT).

1. Asthma/COPD

We reviewed the literature on asthma/COPD incidence models and did not find a robust risk prediction model for Middle Eastern populations. Hence, we constructed a multivariate risk score for asthma or COPD morbidity (moderate to severe exacerbation) risk by age, utilizing relative risk estimates from a literature review. To identify literature for risk factors for asthma/COPD morbidity, we used combinations of the search terms "asthma exacerbations", "COPD exacerbations", "risk factors for asthma exacerbations", "risk factors for copd exacerbations", "smoking", "body mass index", "prior diagnosis", "age", "gender", "race", "ethnicity", "socioeconomic status", "environmental factors", "occupational factors", "medications", "allergies", "asthma severity", "COPD severity", "lung function", "inflammation", "airway hyperresponsiveness", "infection", "stress", "nutrition", "sleep", "psychosocial factors", "physical activity", "genetics", "pharmacogenetic factors", "genetic polymorphisms". We found 15 relevant articles focusing on the key predictors of relative risk of asthma or COPD exacerbations by age and sex [[122,123,123–129,129–134]](https://www.zotero.org/google-docs/?KCf9Ut). Based on the most consistently-reported predictors covering a majority of variance in incidence in the region, constructed an equation for the relative risk of exacerbations as the product of average risk by age and sex, relative risk increase due to tobacco smoking, and relative risk from higher BMI. The age risk by age and sex was obtained from estimates for Palestine from the Global Burden of Disease [[135]](https://www.zotero.org/google-docs/?H7AAYX), while estimates for current smoking were a relative risk of 3.51 (3.08-3.99), for prior smoking were a relative risk of 2.35 (2.11-2.63), and for high BMI was a relative risk of 1.21 (1.16–1.26) for each three unit increase in BMI over 25 kg/m^2 [[126,128]](https://www.zotero.org/google-docs/?byunC5). Among incident cases, we distributed the proportion attributed to asthma versus COPD using the Global Burden of Disease estimates for asthma vs COPD morbidity by age and sex [[135]](https://www.zotero.org/google-docs/?vGXgGz), and applied the subsequent intervention effectiveness to the subset simulated as having asthma specifically.

1. Colorectal cancer

We compared multiple models of colorectal cancer prediction based on a prior meta-analysis [[136]](https://www.zotero.org/google-docs/?aKkcEM), and settled on the CRC-Pro given its more robust discrimination statistics among diverse populations including those from the Middle East. CRC-Pro was developed using data from the Colon Cancer Family Registry, a large cohort study of people with a family history of CRC. The model includes 11 risk factors, including age, family history, personal history of polyps, and lifestyle factors [[137]](https://www.zotero.org/google-docs/?JhInUC). The model was developed using data from the Multi-Ethnic Cohort Study and demonstrated good accuracy with a cross-validated C-statistic of 0.681 in men and 0.679 in women [[137]](https://www.zotero.org/google-docs/?kEoDU3). It has been internally validated in diverse populations but without specific subgroup analyses among Palestinian or Middle-Eastern populations [[136,137]](https://www.zotero.org/google-docs/?hbi2Rs). As with the other risk scores, calibration to the Palestinian population in Gaza was performed by computing each risk score across each individual in the population-representative study sample, then scaling the risk scores to achieve the estimated incidence of each NCD in the Palestinian population from the Global Burden of Disease project [[135]](https://www.zotero.org/google-docs/?OxfaAN).

1. Breast cancer

We compared multiple models of breast cancer prediction including the Gail model [[138]](https://www.zotero.org/google-docs/?hK5Wm9), Tyrer-Cuzick [[139]](https://www.zotero.org/google-docs/?IKUk8i), BOADICEA [[140]](https://www.zotero.org/google-docs/?SnoU90), BCSC [[141]](https://www.zotero.org/google-docs/?gZop5l), Rosner-Colditz [[29]](https://www.zotero.org/google-docs/?Cb6h5H), and Claus models [[142]](https://www.zotero.org/google-docs/?a18oxo). Due to the lack of available of BRCA genetic data or mammographic density data, and the validation of the Gail model in multi-ethnic populations including in the Middle East and South Asia [[143–145]](https://www.zotero.org/google-docs/?ENvC0v), we chose the Gail model over the other alternatives. The Gail model had an AUC of 0.63 among Iranian women [[146]](https://www.zotero.org/google-docs/?rNNtDg), and we did not find validity assessments of the alternative models among Middle Eastern populations.

**Supporting Information References**

[1. Page MJ, McKenzie JE, Bossuyt PM, Boutron I, Hoffmann TC, Mulrow CD, et al. The PRISMA 2020 statement: An updated guideline for reporting systematic reviews. PLOS Med. 2021;18: e1003583. doi:10.1371/journal.pmed.1003583](https://www.zotero.org/google-docs/?kNRxsh)

[2. RoB 2: A revised Cochrane risk-of-bias tool for randomized trials | Cochrane Bias. [cited 5 Jul 2023]. Available: https://methods.cochrane.org/bias/resources/rob-2-revised-cochrane-risk-bias-tool-randomized-trials](https://www.zotero.org/google-docs/?kNRxsh)

[3. ROBINS-I tool | Cochrane Methods. [cited 5 Jul 2023]. Available: https://methods.cochrane.org/robins-i](https://www.zotero.org/google-docs/?kNRxsh)

[4. Edwards R, Thomson G, Wilson N, Waa A, Bullen C, O’Dea D, et al. After the smoke has cleared: evaluation of the impact of a new national smoke-free law in New Zealand. Tob Control. 2008;17: e2–e2. doi:10.1136/tc.2007.020347](https://www.zotero.org/google-docs/?kNRxsh)

[5. Hopkins DP, Razi S, Leeks KD, Kalra GP, Chattopadhyay SK, Soler RE. Smokefree Policies to Reduce Tobacco Use: A Systematic Review. Am J Prev Med. 2010;38: S275–S289. doi:10.1016/j.amepre.2009.10.029](https://www.zotero.org/google-docs/?kNRxsh)

[6. Hyland A, Barnoya J, Corral JE. Smoke-free air policies: past, present and future. Tob Control. 2012;21: 154–161. doi:10.1136/tobaccocontrol-2011-050389](https://www.zotero.org/google-docs/?kNRxsh)

[7. Brownson RC, Eriksen MP, Davis RM, Warner KE. ENVIRONMENTAL TOBACCO SMOKE:Health Effects and Policies to Reduce Exposure. Annu Rev Public Health. 1997;18: 163–185. doi:10.1146/annurev.publhealth.18.1.163](https://www.zotero.org/google-docs/?kNRxsh)

[8. Blanco-Marquizo A, Goja B, Peruga A, Jones MR, Yuan J, Samet JM, et al. Reduction of secondhand tobacco smoke in public places following national smoke-free legislation in Uruguay. Tob Control. 2010;19: 231–234. doi:10.1136/tc.2009.034769](https://www.zotero.org/google-docs/?kNRxsh)

[9. Stillman F, Navas-Acien A, Ma J, Ma S, Avila-Tang E, Breysse P, et al. Second-hand tobacco smoke in public places in urban and rural China. Tob Control. 2007;16: 229–234. doi:10.1136/tc.2006.018333](https://www.zotero.org/google-docs/?kNRxsh)

[10. Ye X, Yao Z, Gao Y, Xu Y, Xu Y, Zhu Z, et al. Second-hand smoke exposure in different types of venues: before and after the implementation of smoke-free legislation in Guangzhou, China. BMJ Open. 2014;4: e004273. doi:10.1136/bmjopen-2013-004273](https://www.zotero.org/google-docs/?kNRxsh)

[11. Ong MK, Glantz SA. Cardiovascular health and economic effects of smoke-free workplaces. Am J Med. 2004;117: 32–38. doi:10.1016/j.amjmed.2004.02.029](https://www.zotero.org/google-docs/?kNRxsh)

[12. Sims M, Maxwell R, Gilmore A. Short-term impact of the smokefree legislation in England on emergency hospital admissions for asthma among adults: a population-based study. Thorax. 2013;68: 619–624. doi:10.1136/thoraxjnl-2012-202841](https://www.zotero.org/google-docs/?kNRxsh)

[13. International Agency for Research on Cancer. Evaluating the Effectiveness of Smoke-free Policies. Geneva: World Health Organization; 2009. Available: https://publications.iarc.fr/Book-And-Report-Series/Iarc-Handbooks-Of-Cancer-Prevention/Evaluating-The-Effectiveness-Of-Smoke-free-Policies-2009](https://www.zotero.org/google-docs/?kNRxsh)

[14. Asaria P, Chisholm D, Mathers C, Ezzati M, Beaglehole R. Chronic disease prevention: health effects and financial costs of strategies to reduce salt intake and control tobacco use. The Lancet. 2007;370: 2044–2053.](https://www.zotero.org/google-docs/?kNRxsh)

[15. Bertram MY, Stenberg K, Brindley C, Li J, Serje J, Watts R, et al. Disease control programme support costs: an update of WHO-CHOICE methodology, price databases and quantity assumptions. Cost Eff Resour Alloc. 2017;15: 21. doi:10.1186/s12962-017-0083-6](https://www.zotero.org/google-docs/?kNRxsh)

[16. Knai C, Pomerleau J, Lock K, McKee M. Getting children to eat more fruit and vegetables: A systematic review. Prev Med. 2006;42: 85–95. doi:10.1016/j.ypmed.2005.11.012](https://www.zotero.org/google-docs/?kNRxsh)

[17. Pomerleau J, Lock K, Knai C, McKee M. Interventions Designed to Increase Adult Fruit and Vegetable Intake Can Be Effective: A Systematic Review of the Literature, ,. J Nutr. 2005;135: 2486–2495. doi:10.1093/jn/135.10.2486](https://www.zotero.org/google-docs/?kNRxsh)

[18. Evans CE, Christian MS, Cleghorn CL, Greenwood DC, Cade JE. Systematic review and meta-analysis of school-based interventions to improve daily fruit and vegetable intake in children aged 5 to 12 y123. Am J Clin Nutr. 2012;96: 889–901. doi:10.3945/ajcn.111.030270](https://www.zotero.org/google-docs/?kNRxsh)

[19. Robinson-O’Brien R, Story M, Heim S. Impact of Garden-Based Youth Nutrition Intervention Programs: A Review - Journal of the American Dietetic Association. J Am Acad Nutr Diet. 2009;109: 273–80.](https://www.zotero.org/google-docs/?kNRxsh)

[20. Anderson AS, Porteous LEG, Foster E, Higgins C, Stead M, Hetherington M, et al. The impact of a school-based nutrition education intervention on dietary intake and cognitive and attitudinal variables relating to fruits and vegetables. Public Health Nutr. 2005;8: 650–656. doi:10.1079/PHN2004721](https://www.zotero.org/google-docs/?kNRxsh)

[21. Aloia CR, Shockey TA, Nahar VK, Knight KB. Pertinence of the recent school-based nutrition interventions targeting fruit and vegetable consumption in the United States:a systematic review. Health Promot Perspect. 2016;6: 1–9. doi:10.15171/hpp.2016.01](https://www.zotero.org/google-docs/?kNRxsh)

[22. Touyz LM, Wakefield CE, Grech AM, Quinn VF, Costa DSJ, Zhang FF, et al. Parent-targeted home-based interventions for increasing fruit and vegetable intake in children: a systematic review and meta-analysis. Nutr Rev. 2018;76: 154–173. doi:10.1093/nutrit/nux066](https://www.zotero.org/google-docs/?kNRxsh)

[23. Herman DR, Harrison GG, Afifi AA, Jenks E. Effect of a targeted subsidy on intake of fruits and vegetables among low-income women in the Special Supplemental Nutrition Program for Women, Infants, and Children. Am J Public Health. 2008;98: 98–105. doi:10.2105/AJPH.2005.079418](https://www.zotero.org/google-docs/?kNRxsh)

[24. Anderson AS, Cox DN, McKellar S, Reynolds J, Lean MEJ, Mela DJ. Take Five, a nutrition education intervention to increase fruit and vegetable intakes: impact on attitudes towards dietary change. Br J Nutr. 1998;80: 133–140. doi:10.1017/S0007114598001032](https://www.zotero.org/google-docs/?kNRxsh)

[25. Bandoni DH, Sarno F, Jaime PC. Impact of an intervention on the availability and consumption of fruits and vegetables in the workplace. Public Health Nutr. 2011;14: 975–981. doi:10.1017/S1368980010003460](https://www.zotero.org/google-docs/?kNRxsh)

[26. Bhiri S, Maatoug J, Zammit N, Msakni Z, Harrabi I, Amimi S, et al. A 3-Year Workplace-Based Intervention Program to Control Noncommunicable Disease Risk Factors in Sousse, Tunisia. J Occup Environ Med. 2015;57: e72. doi:10.1097/JOM.0000000000000500](https://www.zotero.org/google-docs/?kNRxsh)

[27. Bazzano LA, Serdula MK, Liu S. Dietary intake of fruits and vegetables and risk of cardiovascular disease. Curr Atheroscler Rep. 2003;5: 492–499. doi:10.1007/s11883-003-0040-z](https://www.zotero.org/google-docs/?kNRxsh)

[28. Carter P, Gray LJ, Troughton J, Khunti K, Davies MJ. Fruit and vegetable intake and incidence of type 2 diabetes mellitus: systematic review and meta-analysis. BMJ. 2010;341: c4229. doi:10.1136/bmj.c4229](https://www.zotero.org/google-docs/?kNRxsh)

[29. Farvid MS, Chen WY, Rosner BA, Tamimi RM, Willett WC, Eliassen AH. Fruit and vegetable consumption and breast cancer incidence: Repeated measures over 30 years of follow-up. Int J Cancer. 2019;144: 1496–1510. doi:10.1002/ijc.31653](https://www.zotero.org/google-docs/?kNRxsh)

[30. Wu Z-Y, Chen J-L, Li H, Su K, Han Y-W. Different types of fruit intake and colorectal cancer risk: A meta-analysis of observational studies. World J Gastroenterol. 2023;29: 2679–2700. doi:10.3748/wjg.v29.i17.2679](https://www.zotero.org/google-docs/?kNRxsh)

[31. Fitzgerald S, Kirby A, Murphy A, Geaney F, Perry IJ. A cost-analysis of complex workplace nutrition education and environmental dietary modification interventions. BMC Public Health. 2017;17: 49. doi:10.1186/s12889-016-3988-7](https://www.zotero.org/google-docs/?kNRxsh)

[32. Brambila-Macias J, Shankar B, Capacci S, Mazzocchi M, Perez-Cueto FJA, Verbeke W, et al. Policy Interventions to Promote Healthy Eating: A Review of What Works, What Does Not, and What is Promising. Food Nutr Bull. 2011;32: 365–375. doi:10.1177/156482651103200408](https://www.zotero.org/google-docs/?kNRxsh)

[33. Wakefield MA, Loken B, Hornik RC. Use of mass media campaigns to change health behaviour. The Lancet. 2010;376: 1261–1271. doi:10.1016/S0140-6736(10)60809-4](https://www.zotero.org/google-docs/?kNRxsh)

[34. Thow AM, Downs S, Jan S. A systematic review of the effectiveness of food taxes and subsidies to improve diets: Understanding the recent evidence. Nutr Rev. 2014;72: 551–565. doi:10.1111/nure.12123](https://www.zotero.org/google-docs/?kNRxsh)

[35. Rekhy R, McConchie R. Promoting consumption of fruit and vegetables for better health. Have campaigns delivered on the goals? Appetite. 2014;79: 113–123. doi:10.1016/j.appet.2014.04.012](https://www.zotero.org/google-docs/?kNRxsh)

[36. Robinson MN, Tansil KA, Elder RW, Soler RE, Labre MP, Mercer SL, et al. Mass Media Health Communication Campaigns Combined with Health-Related Product Distribution: A Community Guide Systematic Review. Am J Prev Med. 2014;47: 360–371. doi:10.1016/j.amepre.2014.05.034](https://www.zotero.org/google-docs/?kNRxsh)

[37. Hyseni L, Atkinson M, Bromley H, Orton L, Lloyd-Williams F, McGill R, et al. The effects of policy actions to improve population dietary patterns and prevent diet-related non-communicable diseases: scoping review. Eur J Clin Nutr. 2017;71: 694–711. doi:10.1038/ejcn.2016.234](https://www.zotero.org/google-docs/?kNRxsh)

[38. Afshin A, Abioye AI, Ajala ON, Nguyen AB, See KC, Mozaffarian D. Abstract P087: Effectiveness of Mass Media Campaigns for Improving Dietary Behaviors: A Systematic Review and Meta-analysis. Circulation. 2013;127: AP087–AP087. doi:10.1161/circ.127.suppl_12.AP087](https://www.zotero.org/google-docs/?kNRxsh)

[39. Murray CJ, Lauer JA, Hutubessy RC, Niessen L, Tomijima N, Rodgers A, et al. Effectiveness and costs of interventions to lower systolic blood pressure and cholesterol: a global and regional analysis on reduction of cardiovascular-disease risk. The Lancet. 2003;361: 717–725. doi:10.1016/S0140-6736(03)12655-4](https://www.zotero.org/google-docs/?kNRxsh)

[40. Iverson D, Fielding J, Crow R, Christenson G. The promotion of physical activity in the United States population: the status of programs in medical, worksite, community, and school settings. Public Health Rep. 1985 [cited 11 Jul 2023]. Available: https://www.semanticscholar.org/paper/The-promotion-of-physical-activity-in-the-United-of-Iverson-Fielding/153c4a65c9f6f892f0262afc492fc14f8b24d432](https://www.zotero.org/google-docs/?kNRxsh)

[41. Sallis JF, Floyd MF, Rodríguez DA, Saelens BE. Role of Built Environments in Physical Activity, Obesity, and Cardiovascular Disease. Circulation. 2012;125: 729–737. doi:10.1161/CIRCULATIONAHA.110.969022](https://www.zotero.org/google-docs/?kNRxsh)

[42. Daniel H, Bornstein SS, Kane GC, Health and Public Policy Committee of the American College of Physicians, Carney JK, Gantzer HE, et al. Addressing Social Determinants to Improve Patient Care and Promote Health Equity: An American College of Physicians Position Paper. Ann Intern Med. 2018;168: 577–578. doi:10.7326/M17-2441](https://www.zotero.org/google-docs/?kNRxsh)

[43. Ramalingam N, Strayer TE, Breig SA, Harden S. How Are Community Health Workers Trained to Deliver Physical Activity to Adults? A Scoping Review. Transl J ACSM. 2019 [cited 11 Jul 2023]. Available: https://www.semanticscholar.org/paper/How-Are-Community-Health-Workers-Trained-to-Deliver-Ramalingam-Strayer/eff63db5160602fa55cbc5832d4817cf44e781e1](https://www.zotero.org/google-docs/?kNRxsh)

[44. Community Preventive Services Task Force. Physical Activity: Community-Wide Campaigns | The Community Guide. Atlanta: CDC; 2023. Available: https://www.thecommunityguide.org/findings/physical-activity-community-wide-campaigns.html](https://www.zotero.org/google-docs/?kNRxsh)

[45. Bull FC, Al-Ansari SS, Biddle S, Borodulin K, Buman MP, Cardon G, et al. World Health Organization 2020 guidelines on physical activity and sedentary behaviour. Br J Sports Med. 2020;54: 1451–1462. doi:10.1136/bjsports-2020-102955](https://www.zotero.org/google-docs/?kNRxsh)

[46. Li J, Siegrist J. Physical Activity and Risk of Cardiovascular Disease—A Meta-Analysis of Prospective Cohort Studies. Int J Environ Res Public Health. 2012;9: 391. doi:10.3390/ijerph9020391](https://www.zotero.org/google-docs/?kNRxsh)

[47. Jeon CY, Lokken RP, Hu FB, van Dam RM. Physical Activity of Moderate Intensity and Risk of Type 2 Diabetes: A systematic review. Diabetes Care. 2007;30: 744–752. doi:10.2337/dc06-1842](https://www.zotero.org/google-docs/?kNRxsh)

[48. Jaakkola JJK, Aalto SAM, Hernberg S, Kiihamäki S-P, Jaakkola MS. Regular exercise improves asthma control in adults: A randomized controlled trial. Sci Rep. 2019;9: 12088. doi:10.1038/s41598-019-48484-8](https://www.zotero.org/google-docs/?kNRxsh)

[49. Garcia-Aymerich J, Lange P, Benet M, Schnohr P, Antó JM. Regular physical activity reduces hospital admission and mortality in chronic obstructive pulmonary disease: a population based cohort study. Thorax. 2006;61: 772–778. doi:10.1136/thx.2006.060145](https://www.zotero.org/google-docs/?kNRxsh)

[50. Guo W, Fensom GK, Reeves GK, Key TJ. Physical activity and breast cancer risk: results from the UK Biobank prospective cohort. Br J Cancer. 2020;122: 726–732. doi:10.1038/s41416-019-0700-6](https://www.zotero.org/google-docs/?kNRxsh)

[51. Wolin KY, Yan Y, Colditz GA, Lee I-M. Physical activity and colon cancer prevention: a meta-analysis. Br J Cancer. 2009;100: 611–616. doi:10.1038/sj.bjc.6604917](https://www.zotero.org/google-docs/?kNRxsh)

[52. Oloo MO, Wamukoya EK, Wanzala M. EFFICACY OF PHYSICAL ACTIVITY COUNSELLING INTERVENTIONS DELIVERED IN PRIMARY CARE: A SYSTEMATIC REVIEW AND META-ANALYSIS. Eur J Phys Educ Sport Sci. 2020;6. doi:10.5281/zenodo.3864522](https://www.zotero.org/google-docs/?kNRxsh)

[53. Quattrin T, Wilfley DE. The Promise and Opportunities for Screening and Treating Childhood Obesity: USPSTF Recommendation Statement. JAMA Pediatr. 2017;171: 733–735. doi:10.1001/jamapediatrics.2017.1604](https://www.zotero.org/google-docs/?kNRxsh)

[54. Curry SJ, Grossman DC, Whitlock EP, Cantu A. Behavioral Counseling Research and Evidence-Based Practice Recommendations: U.S. Preventive Services Task Force Perspectives. Ann Intern Med. 2014;160: 407–413. doi:10.7326/M13-2128](https://www.zotero.org/google-docs/?kNRxsh)

[55. Lin JS, O’Connor E, Evans CV, Senger CA, Rowland MG, Groom HC. Behavioral Counseling to Promote a Healthy Lifestyle in Persons With Cardiovascular Risk Factors: A Systematic Review for the U.S. Preventive Services Task Force. Ann Intern Med. 2014;161: 568–578. doi:10.7326/M14-0130](https://www.zotero.org/google-docs/?kNRxsh)

[56. Aubert RE, Herman WH, Waters J, Moore W, Sutton D, Peterson BL, et al. Nurse Case Management To Improve Glycemic Control in Diabetic Patients in a Health Maintenance Organization. Ann Intern Med. 1998;129: 605–612. doi:10.7326/0003-4819-129-8-199810150-00004](https://www.zotero.org/google-docs/?kNRxsh)

[57. Hogg WE, Zhao X, Angus D, Fortier M, Zhong J, O’Sullivan T, et al. The Cost of Integrating a Physical Activity Counselor in the Primary Health Care Team. J Am Board Fam Med. 2012;25: 250–252. doi:10.3122/jabfm.2012.02.110154](https://www.zotero.org/google-docs/?kNRxsh)

[58. Salzmann P, Kerlikowske K, Phillips K. Cost-Effectiveness of Extending Screening Mammography Guidelines To Include Women 40 to 49 Years of Age. Ann Intern Med. 1997;127: 955–965. doi:10.7326/0003-4819-127-11-199712010-00001](https://www.zotero.org/google-docs/?kNRxsh)

[59. Nattinger AB, Mitchell JL. Breast Cancer Screening and Prevention. Ann Intern Med. 2016;164: ITC81–ITC96. doi:10.7326/AITC201606070](https://www.zotero.org/google-docs/?kNRxsh)

[60. Miller RG. Breast cancer screening. J Gen Intern Med. 2001;16: 206–207. doi:10.1111/j.1525-1497.2001.10119.x](https://www.zotero.org/google-docs/?kNRxsh)

[61. Elmore J, Choe J. Breast Cancer Screening for Women in Their 40s: Moving from Controversy about Data to Helping Individual Women. Ann Intern Med. 2007;146: 529–531. doi:10.7326/0003-4819-146-7-200704030-00010](https://www.zotero.org/google-docs/?kNRxsh)

[62. Tabár L, Vitak B, Chen TH-H, Yen AM-F, Cohen A, Tot T, et al. Swedish Two-County Trial: Impact of Mammographic Screening on Breast Cancer Mortality during 3 Decades. Radiology. 2011;260: 658–663. doi:10.1148/radiol.11110469](https://www.zotero.org/google-docs/?kNRxsh)

[63. Broeders M, Moss S, Nyström L, Njor S, Jonsson H, Paap E, et al. The Impact of Mammographic Screening on Breast Cancer Mortality in Europe: A Review of Observational Studies. J Med Screen. 2012;19: 14–25. doi:10.1258/jms.2012.012078](https://www.zotero.org/google-docs/?kNRxsh)

[64. Weedon-Fekjær H, Romundstad PR, Vatten LJ. Modern mammography screening and breast cancer mortality: population study. BMJ. 2014;348: g3701. doi:10.1136/bmj.g3701](https://www.zotero.org/google-docs/?kNRxsh)

[65. Nickson C, Mason KE, English DR, Kavanagh AM. Mammographic Screening and Breast Cancer Mortality: A Case–Control Study and Meta-analysis. Cancer Epidemiol Biomarkers Prev. 2012;21: 1479–1488. doi:10.1158/1055-9965.EPI-12-0468](https://www.zotero.org/google-docs/?kNRxsh)

[66. Otto SJ, Fracheboud J, Verbeek ALM, Boer R, Reijerink-Verheij JCIY, Otten JDM, et al. Mammography Screening and Risk of Breast Cancer Death: A Population-Based Case–Control Study. Cancer Epidemiol Biomarkers Prev. 2012;21: 66–73. doi:10.1158/1055-9965.EPI-11-0476](https://www.zotero.org/google-docs/?kNRxsh)

[67. Autier P, Koechlin A, Smans M, Vatten L, Boniol M. Mammography Screening and Breast Cancer Mortality in Sweden. JNCI J Natl Cancer Inst. 2012;104: 1080–1093. doi:10.1093/jnci/djs272](https://www.zotero.org/google-docs/?kNRxsh)

[68. Morrell S, Taylor R, Roder D, Dobson A. Mammography screening and breast cancer mortality in Australia: an aggregate cohort study. J Med Screen. 2012;19: 26–34. doi:10.1258/jms.2012.011127](https://www.zotero.org/google-docs/?kNRxsh)

[69. Magnus MC, Ping M, Shen MM, Bourgeois J, Magnus JH. Effectiveness of Mammography Screening in Reducing Breast Cancer Mortality in Women Aged 39–49 Years: A Meta-Analysis. J Womens Health. 2011;20: 845–852. doi:10.1089/jwh.2010.2098](https://www.zotero.org/google-docs/?kNRxsh)

[70. Irvin VL, Kaplan RM. Screening Mammography & Breast Cancer Mortality: Meta-Analysis of Quasi-Experimental Studies. PLOS ONE. 2014;9: e98105. doi:10.1371/journal.pone.0098105](https://www.zotero.org/google-docs/?kNRxsh)

[71. menaadmin. Economical issues hindering breast cancer screening and early detection. In: AIB Middle East and North Africa Chapter [Internet]. 5 May 2021 [cited 13 Jul 2023]. Available: https://mena.aib.world/publications/policy-insights/economical-issues-hindering-breast-cancer-screening-and-early-detection/](https://www.zotero.org/google-docs/?kNRxsh)

[72. Simpson J, Scholefield JH. Treatment of colorectal cancer: surgery, chemotherapy and radiotherapy. Surg - Oxf Int Ed. 2008;26: 329–333. doi:10.1016/j.mpsur.2008.06.003](https://www.zotero.org/google-docs/?kNRxsh)

[73. Tokodai K, Narimatsu H, Nishida A, Takaya K, Hara Y, Kawagishi N, et al. Risk factors for recurrence in stage II/III colorectal cancer patients treated with curative surgery: The impact of postoperative tumor markers and an infiltrative growth pattern. J Surg Oncol. 2016;114: 368–374. doi:10.1002/jso.24320](https://www.zotero.org/google-docs/?kNRxsh)

[74. Simillis C, Singh HKSI, Afxentiou T, Mills S, Warren OJ, Smith JJ, et al. Postoperative chemotherapy improves survival in patients with resected high-risk Stage II colorectal cancer: results of a systematic review and meta-analysis. Colorectal Dis. 2020;22: 1231–1244. doi:10.1111/codi.14994](https://www.zotero.org/google-docs/?kNRxsh)

[75. Link KH, Staib L, Kreuser E-D, Beger HG. Adjuvant Treatment of Colon and Rectal Cancer: Impact of Chemotherapy, Radiotherapy, and Immunotherapy on Routine Postsurgical Patient Management. In: Kreuser E-D, Schlag PM, editors. New Perspectives in Molecular and Clinical Management of Gastrointestinal Tumors. Berlin, Heidelberg: Springer; 1996. pp. 311–352. doi:10.1007/978-3-642-80035-1_19](https://www.zotero.org/google-docs/?kNRxsh)

[76. Wu X, Zhang J, He X, Wang C, Lian L, Liu H, et al. Postoperative Adjuvant Chemotherapy for Stage II Colorectal Cancer: A Systematic Review of 12 Randomized Controlled Trials. J Gastrointest Surg. 2012;16: 646–655. doi:10.1007/s11605-011-1682-8](https://www.zotero.org/google-docs/?kNRxsh)

[77. Johnston PG. Stage II Colorectal Cancer: To Treat or not to Treat. The Oncologist. 2005;10: 332–334. doi:10.1634/theoncologist.10-5-332](https://www.zotero.org/google-docs/?kNRxsh)

[78. Baddi L, Benson A III. Adjuvant Therapy in Stage II Colon Cancer: Current Approaches. The Oncologist. 2005;10: 325–331. doi:10.1634/theoncologist.10-5-325](https://www.zotero.org/google-docs/?kNRxsh)

[79. Rousseau B, Chibaudel B, Bachet J-B, Larsen AK, Tournigand C, Louvet C, et al. Stage II and Stage III Colon Cancer: Treatment Advances and Future Directions. Cancer J. 2010;16: 202. doi:10.1097/PPO.0b013e3181ddc5bf](https://www.zotero.org/google-docs/?kNRxsh)

[80. Chau I, Chan S, Cunningham D. Overview of Preoperative and Postoperative Therapy for Colorectal Cancer: The European and United States Perspectives. Clin Colorectal Cancer. 2003;3: 19–33. doi:10.3816/CCC.2003.n.009](https://www.zotero.org/google-docs/?kNRxsh)

[81. Marshall J. Risk assessment in Stage II colorectal cancer. Oncology. 2010 [cited 20 Jul 2023]. Available: https://www.semanticscholar.org/paper/Risk-assessment-in-Stage-II-colorectal-cancer.-Marshall/8436d2e24dd538b2324dd347d4c24ce17788bd06](https://www.zotero.org/google-docs/?kNRxsh)

[82. American Society of Clinical Oncology. Colorectal Cancer: Survivorship. Alexandria, VA: ASCO; 2012. Available: https://www.cancer.net/cancer-types/colorectal-cancer/statistics](https://www.zotero.org/google-docs/?kNRxsh)

[83. Alefan Q, Malhees R, Mhaidat N. Direct medical cost associated with colorectal cancer in north of Jordan. Curr Probl Cancer. 2017;41: 371–381. doi:10.1016/j.currproblcancer.2017.05.001](https://www.zotero.org/google-docs/?kNRxsh)

[84. Skelton M, Alameddine R, Saifi O, Hammoud M, Zorkot M, Daher M, et al. High-Cost Cancer Treatment Across Borders in Conflict Zones: Experience of Iraqi Patients in Lebanon. JCO Glob Oncol. 2020; 59–66. doi:10.1200/JGO.19.00281](https://www.zotero.org/google-docs/?kNRxsh)

[85. Alghamdi A, Balkhi B, Alqahtani S, Almotairi H. The Economic Burden Associated with the Management of Different Stages of Breast Cancer: A Retrospective Cost of Illness Analysis in Saudi Arabia. Healthcare. 2021;9: 907. doi:10.3390/healthcare9070907](https://www.zotero.org/google-docs/?kNRxsh)

[86. REZAPOUR A, NARGESI S, MEZGINEJAD F, RASHKI KEMMAK A, BAGHERZADEH R. The Economic Burden of Cancer in Iran during 1995–2019: A Systematic Review. Iran J Public Health. 2021;50: 35–45. doi:10.18502/ijph.v50i1.5070](https://www.zotero.org/google-docs/?kNRxsh)

[87. Goodman M. Adjuvant systemic therapy of stage I and II breast cancer. Semin Oncol Nurs. 1991;7: 175–186. doi:10.1016/0749-2081(91)90030-S](https://www.zotero.org/google-docs/?kNRxsh)

[88. Blum JL, Jones SE, Fay JW, Senzer N, Mennel RG. Guidelines for systemic therapy of early stage breast cancer. Breast Cancer Res Treat. 1997;43: 259–276. doi:10.1023/A:1005705300012](https://www.zotero.org/google-docs/?kNRxsh)

[89. Newman LA, Singletary SE. Overview of Adjuvant Systemic Therapy in Early Stage Breast Cancer. Surg Clin North Am. 2007;87: 499–509. doi:10.1016/j.suc.2007.01.002](https://www.zotero.org/google-docs/?kNRxsh)

[90. Howell A, Wardley AM. Overview of the impact of conventional systemic therapies on breast cancer. Endocr Relat Cancer. 2005;12: S9–S16. doi:10.1677/erc.1.01003](https://www.zotero.org/google-docs/?kNRxsh)

[91. Guarneri V, Frassoldati A, Giovannelli S, Borghi F, Conte P. Primary systemic therapy for operable breast cancer: A review of clinical trials and perspectives. Cancer Lett. 2007;248: 175–185. doi:10.1016/j.canlet.2006.07.001](https://www.zotero.org/google-docs/?kNRxsh)

[92. Bonadonna G, Valagussa P. Systemic Therapy in Resectable Breast Cancer. Hematol Oncol Clin North Am. 1989;3: 727–742. doi:10.1016/S0889-8588(18)30529-X](https://www.zotero.org/google-docs/?kNRxsh)

[93. Khan SA, Stewart AK, Morrow M. Does aggressive local therapy improve survival in metastatic breast cancer? Surgery. 2002;132: 620–627. doi:10.1067/msy.2002.127544](https://www.zotero.org/google-docs/?kNRxsh)

[94. iety. Breast Cancer Facts & Figures. Kennesaw, GA: ACS; 2023. Available: https://www.cancer.org/research/cancer-facts-statistics/breast-cancer-facts-figures.html](https://www.zotero.org/google-docs/?kNRxsh)

[95. Busse WW, Brazinsky S, Jacobson K, Stricker W, Schmitt K, Burgt JV, et al. Efficacy response of inhaled beclomethasone dipropionate in asthma is proportional to dose and is improved by formulation with a new propellant. J Allergy Clin Immunol. 1999;104: 1215–1222. doi:10.1016/S0091-6749(99)70016-3](https://www.zotero.org/google-docs/?kNRxsh)

[96. Clark TJH. EFFECT OF BECLOMETHASONE DIPROPIONATE DELIVERED BY AEROSOL IN PATIENTS WITH ASTHMA. The Lancet. 1972;299: 1361–1364. doi:10.1016/S0140-6736(72)91094-X](https://www.zotero.org/google-docs/?kNRxsh)

[97. Huchon G, Magnussen H, Chuchalin A, Dymek L, Gonod FB, Bousquet J. Lung function and asthma control with beclomethasone and formoterol in a single inhaler. Respir Med. 2009;103: 41–49. doi:10.1016/j.rmed.2008.09.002](https://www.zotero.org/google-docs/?kNRxsh)

[98. Spears M, Donnelly I, Jolly L, Brannigan M, Ito K, McSharry C, et al. Effect of low-dose theophylline plus beclometasone on lung function in smokers with asthma: a pilot study. Eur Respir J. 2009;33: 1010–1017. doi:10.1183/09031936.00158208](https://www.zotero.org/google-docs/?kNRxsh)

[99. Smith MJ, Hodson M. HIGH-DOSE BECLOMETHASONE INHALER IN THE TREATMENT OF ASTHMA. The Lancet. 1983;321: 265–269. doi:10.1016/S0140-6736(83)91686-0](https://www.zotero.org/google-docs/?kNRxsh)

[100. Papi A, Corradi M, Pigeon-Francisco C, Baronio R, Siergiejko Z, Petruzzelli S, et al. Beclometasone–formoterol as maintenance and reliever treatment in patients with asthma: a double-blind, randomised controlled trial. Lancet Respir Med. 2013;1: 23–31. doi:10.1016/S2213-2600(13)70012-2](https://www.zotero.org/google-docs/?kNRxsh)

[101. Fahy JV, Boushey HA. Effect of low-dose beclomethasone dipropionate on asthma control and airway inflammation. Eur Respir J. 1998;11: 1240–1247. doi:10.1183/09031936.98.11061240](https://www.zotero.org/google-docs/?kNRxsh)

[102. Gagnon M, Côte J, Milot J, Turcotte H, Boulet L-P. Comparative Safety and Efficacy of Single or Twice Daily Administration of Inhaled Beclomethasone in Moderate Asthma. CHEST. 1994;105: 1732–1737. doi:10.1378/chest.105.6.1732](https://www.zotero.org/google-docs/?kNRxsh)

[103. Fabbri LM, Nicolini G, Olivieri D, Papi A. Inhaled beclometasone dipropionate/formoterol extra-fine fixed combination in the treatment of asthma: evidence and future perspectives. Expert Opin Pharmacother. 2008;9: 479–490. doi:10.1517/14656566.9.3.479](https://www.zotero.org/google-docs/?kNRxsh)

[104. Brusselle G, Nicolini G, Santoro L, Guastalla D, Papi A. Beclometasone dipropionate/formoterol maintenance and reliever therapy asthma exacerbation benefit increases with blood eosinophil level. Eur Respir J. 2021;58. doi:10.1183/13993003.040982020](https://www.zotero.org/google-docs/?kNRxsh)

[105. Hatter L, Bruce P, Braithwaite I, Holliday M, Fingleton J, Weatherall M, et al. ICS-formoterol reliever versus ICS and short-acting β2-agonist reliever in asthma: a systematic review and meta-analysis. ERJ Open Res. 2021;7. doi:10.1183/23120541.00701-2020](https://www.zotero.org/google-docs/?kNRxsh)

[106. Al Mazrouei K, Almannaei AI, Nur FM, Bachnak N, Alzaabi A. Direct and Indirect Costs of Asthma Burden in Abu Dhabi: A Retrospective Analysis of Insurance Claims Data from 2015 to 2018. Clin Outcomes Res CEOR. 2021;13: 969–980. doi:10.2147/CEOR.S331499](https://www.zotero.org/google-docs/?kNRxsh)

[107. Alzaabi A, Alseiari M, Mahboub B. Economic burden of asthma in Abu Dhabi: a retrospective study. Clin Outcomes Res CEOR. 2014;6: 445–450. doi:10.2147/CEOR.S68920](https://www.zotero.org/google-docs/?kNRxsh)

[108. Wilson PW, D’Agostino RB, Levy D, Belanger AM, Silbershatz H, Kannel WB. Prediction of coronary heart disease using risk factor categories. Circulation. 1998;97: 1837–1847. doi:10.1161/01.cir.97.18.1837](https://www.zotero.org/google-docs/?kNRxsh)

[109. Mendis S, Lindholm LH, Mancia G, Whitworth J, Alderman M, Lim S, et al. World Health Organization (WHO) and International Society of Hypertension (ISH) risk prediction charts: assessment of cardiovascular risk for prevention and control of cardiovascular disease in low and middle-income countries. J Hypertens. 2007;25: 1578–1582.](https://www.zotero.org/google-docs/?kNRxsh)

[110. Goff DC, Lloyd-Jones DM, Bennett G, Coady S, D’Agostino RB, Gibbons R, et al. 2013 ACC/AHA Guideline on the Assessment of Cardiovascular Risk. Circulation. 2014;129: S49–S73. doi:10.1161/01.cir.0000437741.48606.98](https://www.zotero.org/google-docs/?kNRxsh)

[111. Hajifathalian K, Ueda P, Lu Y, Woodward M, Ahmadvand A, Aguilar-Salinas CA, et al. A novel risk score to predict cardiovascular disease risk in national populations (Globorisk): a pooled analysis of prospective cohorts and health examination surveys. Lancet Diabetes Endocrinol. 2015;3: 339–355. doi:10.1016/S2213-8587(15)00081-9](https://www.zotero.org/google-docs/?kNRxsh)

[112. Ueda P, Woodward M, Lu Y, Hajifathalian K, Al-Wotayan R, Aguilar-Salinas CA, et al. Laboratory-based and office-based risk scores and charts to predict 10-year risk of cardiovascular disease in 182 countries: a pooled analysis of prospective cohorts and health surveys. Lancet Diabetes Endocrinol. 2017;5: 196–213. doi:10.1016/S2213-8587(17)30015-3](https://www.zotero.org/google-docs/?kNRxsh)

[113. Jahangiry L, Dehghan A, Farjam M, Aune D, Rezaei F. Laboratory-based and office-based Globorisk scores to predict 10-year risk of cardiovascular diseases among Iranians: results from the Fasa PERSIAN cohort. BMC Med Res Methodol. 2022;22: 305. doi:10.1186/s12874-022-01791-7](https://www.zotero.org/google-docs/?kNRxsh)

[114. Boyer C. globorisk: R Package for Globorisk Global CVD Risk Calculator. 26 May 2022 [cited 24 Jul 2023]. Available: https://github.com/boyercb/globorisk](https://www.zotero.org/google-docs/?kNRxsh)

[115. Chen L, Magliano DJ, Balkau B, Colagiuri S, Zimmet PZ, Tonkin AM, et al. AUSDRISK: an Australian Type 2 Diabetes Risk Assessment Tool based on demographic, lifestyle and simple anthropometric measures. Med J Aust. 2010;192: 197–202. doi:10.5694/j.1326-5377.2010.tb03507.x](https://www.zotero.org/google-docs/?kNRxsh)

[116. Stern MP, Williams K, Haffner SM. Identification of persons at high risk for type 2 diabetes mellitus: do we need the oral glucose tolerance test? Ann Intern Med. 2002;136: 575–581. doi:10.7326/0003-4819-136-8-200204160-00006](https://www.zotero.org/google-docs/?kNRxsh)

[117. Schmidt MI, Duncan BB, Bang H, Pankow JS, Ballantyne CM, Golden SH, et al. Identifying individuals at high risk for diabetes: The Atherosclerosis Risk in Communities study. Diabetes Care. 2005;28: 2013–2018. doi:10.2337/diacare.28.8.2013](https://www.zotero.org/google-docs/?kNRxsh)

[118. Lindström J, Tuomilehto J. The diabetes risk score: a practical tool to predict type 2 diabetes risk. Diabetes Care. 2003;26: 725–731. doi:10.2337/diacare.26.3.725](https://www.zotero.org/google-docs/?kNRxsh)

[119. Abdallah M, Sharbaji S, Sharbaji M, Daher Z, Faour T, Mansour Z, et al. Diagnostic accuracy of the Finnish Diabetes Risk Score for the prediction of undiagnosed type 2 diabetes, prediabetes, and metabolic syndrome in the Lebanese University. Diabetol Metab Syndr. 2020;12: 84. doi:10.1186/s13098-020-00590-8](https://www.zotero.org/google-docs/?kNRxsh)

[120. Makrilakis K, Liatis S, Grammatikou S, Perrea D, Stathi C, Tsiligros P, et al. Validation of the Finnish diabetes risk score (FINDRISC) questionnaire for screening for undiagnosed type 2 diabetes, dysglycaemia and the metabolic syndrome in Greece. Diabetes Metab. 2011;37: 144–151. doi:10.1016/j.diabet.2010.09.006](https://www.zotero.org/google-docs/?kNRxsh)

[121. Soriguer F, Valdés S, Tapia MJ, Esteva I, Ruiz de Adana MS, Almaraz MC, et al. [Validation of the FINDRISC (FINnish Diabetes RIsk SCore) for prediction of the risk of type 2 diabetes in a population of southern Spain. Pizarra Study]. Med Clin (Barc). 2012;138: 371–376. doi:10.1016/j.medcli.2011.05.025](https://www.zotero.org/google-docs/?kNRxsh)

[122. Huovinen E, Kaprio J, Koskenvuo M. Factors associated to lifestyle and risk of adult onset asthma. Respir Med. 2003;97: 273–280. doi:10.1053/rmed.2003.1419](https://www.zotero.org/google-docs/?kNRxsh)

[123. King M, Mannino D, Holguin F. Risk factors for asthma incidence. A review of recent prospective evidence. Panminerva Med. 2004 [cited 30 Jul 2023]. Available: https://www.semanticscholar.org/paper/Risk-factors-for-asthma-incidence.-A-review-of-King-Mannino/a83a5f21258d409dbf0c8bb699f00f950b583a92](https://www.zotero.org/google-docs/?kNRxsh)

[124. Ronmark E. Obesity increases the risk of incident asthma among adults. Eur Respir J. 2005;25: 282–288. doi:10.1183/09031936.05.00054304](https://www.zotero.org/google-docs/?kNRxsh)

[125. Bahadori K, FitzGerald J. Risk factors of hospitalization and readmission of patients with COPD exacerbation – systematic review. Int J Chron Obstruct Pulmon Dis. 2007 [cited 30 Jul 2023]. Available: https://www.semanticscholar.org/paper/Risk-factors-of-hospitalization-and-readmission-of-Bahadori-FitzGerald/88c5c16efbb47ac7e66d32b3964f935d5f4ac84b](https://www.zotero.org/google-docs/?kNRxsh)

[126. Hjellvik V, Tverdal A, Furu K. Body mass index as predictor for asthma: a cohort study of 118,723 males and females. Eur Respir J. 2010;35: 1235–1242. doi:10.1183/09031936.00192408](https://www.zotero.org/google-docs/?kNRxsh)

[127. Ciprandi G, Ruffoni S, Tosca M, Minetti I, Dellepiane S. Asthma and COPD exacerbations: An 8year survey. Eur J Intern Med. 2011;22: e9–e11. doi:10.1016/j.ejim.2010.11.015](https://www.zotero.org/google-docs/?kNRxsh)

[128. Forey BA, Thornton AJ, Lee PN. Systematic review with meta-analysis of the epidemiological evidence relating smoking to COPD, chronic bronchitis and emphysema. BMC Pulm Med. 2011;11: 36. doi:10.1186/1471-2466-11-36](https://www.zotero.org/google-docs/?kNRxsh)

[129. Al-ani S, Spigt M, Hofset P, Melbye H. Predictors of exacerbations of asthma and COPD during one year in primary care. Fam Pract. 2013;30: 621–628. doi:10.1093/fampra/cmt055](https://www.zotero.org/google-docs/?kNRxsh)

[130. Schatz M, Zeiger RS, Zhang F, Chen W, Yang S-J, Camargo CA. Overweight/Obesity and Risk of Seasonal Asthma Exacerbations. J Allergy Clin Immunol Pract. 2013;1: 618–622. doi:10.1016/j.jaip.2013.07.009](https://www.zotero.org/google-docs/?kNRxsh)

[131. Montserrat-Capdevila J, Godoy P, Marsal JR, Barbé F, Galván L. Risk factors for exacerbation in chronic obstructive pulmonary disease: a prospective study. Int J Tuberc Lung Dis. 2016;20: 389–395. doi:10.5588/ijtld.15.0441](https://www.zotero.org/google-docs/?kNRxsh)

[132. Patra J, Maher YI, Mishra S, Bhatia M, Alam D, Malini DS, et al. Effects of body mass index, tobacco smoking, alcohol drinking and solid fuel use on the risk of asthma: Individual Participant Data (IPD) meta-analysis of 175 000 individuals from 51 nationally representative surveys. BMJ Open Respir Res. 2016;3: e000121. doi:10.1136/bmjresp-2015-000121](https://www.zotero.org/google-docs/?kNRxsh)

[133. Mantero M, Rogliani P, Di Pasquale M, Polverino E, Crisafulli E, Guerrero M, et al. Acute exacerbations of COPD: risk factors for failure and relapse. Int J Chron Obstruct Pulmon Dis. 2017;Volume 12: 2687–2693. doi:10.2147/COPD.S145253](https://www.zotero.org/google-docs/?kNRxsh)

[134. Hogea S, Tudorache E, Fildan AP, Fira‐Mladinescu O, Marc M, Oancea C. Risk factors of chronic obstructive pulmonary disease exacerbations. Clin Respir J. 2020;14: 183–197. doi:10.1111/crj.13129](https://www.zotero.org/google-docs/?kNRxsh)

[135. Institute for Health Metrics and Evaluation. GBD Compare. Seattle: University of Washington; 2023. Available: http://vizhub.healthdata.org/gbd-compare](https://www.zotero.org/google-docs/?kNRxsh)

[136. Usher-Smith JA, Harshfield A, Saunders CL, Sharp SJ, Emery J, Walter FM, et al. External validation of risk prediction models for incident colorectal cancer using UK Biobank. Br J Cancer. 2018;118: 750–759. doi:10.1038/bjc.2017.463](https://www.zotero.org/google-docs/?kNRxsh)

[137. Wells BJ, Kattan MW, Cooper GS, Jackson L, Koroukian S. ColoRectal Cancer Predicted Risk Online (CRC-PRO) Calculator Using Data from the Multi-Ethnic Cohort Study. J Am Board Fam Med. 2014;27: 42–55. doi:10.3122/jabfm.2014.01.130040](https://www.zotero.org/google-docs/?kNRxsh)

[138. Gail MH, Brinton LA, Byar DP, Corle DK, Green SB, Schairer C, et al. Projecting individualized probabilities of developing breast cancer for white females who are being examined annually. J Natl Cancer Inst. 1989;81: 1879–1886. doi:10.1093/jnci/81.24.1879](https://www.zotero.org/google-docs/?kNRxsh)

[139. Tyrer J, Duffy SW, Cuzick J. A breast cancer prediction model incorporating familial and personal risk factors. Stat Med. 2004;23: 1111–1130. doi:10.1002/sim.1668](https://www.zotero.org/google-docs/?kNRxsh)

[140. Lee A, Mavaddat N, Wilcox AN, Cunningham AP, Carver T, Hartley S, et al. BOADICEA: a comprehensive breast cancer risk prediction model incorporating genetic and nongenetic risk factors. Genet Med. 2019;21: 1708–1718. doi:10.1038/s41436-018-0406-9](https://www.zotero.org/google-docs/?kNRxsh)

[141. Tice JA, Cummings SR, Smith-Bindman R, Ichikawa L, Barlow WE, Kerlikowske K. Using Clinical Factors and Mammographic Breast Density to Estimate Breast Cancer Risk: Development and Validation of a New Predictive Model. Ann Intern Med. 2008;148: 337–347.](https://www.zotero.org/google-docs/?kNRxsh)

[142. Fischer C, Kuchenbäcker K, Engel C, Zachariae S, Rhiem K, Meindl A, et al. Evaluating the performance of the breast cancer genetic risk models BOADICEA, IBIS, BRCAPRO and Claus for predicting BRCA1/2 mutation carrier probabilities: a study based on 7352 families from the German Hereditary Breast and Ovarian Cancer Consortium. J Med Genet. 2013;50: 360–367. doi:10.1136/jmedgenet-2012-101415](https://www.zotero.org/google-docs/?kNRxsh)

[143. Costantino JP, Gail MH, Pee D, Anderson S, Redmond CK, Benichou J, et al. Validation studies for models projecting the risk of invasive and total breast cancer incidence. J Natl Cancer Inst. 1999;91: 1541–1548. doi:10.1093/jnci/91.18.1541](https://www.zotero.org/google-docs/?kNRxsh)

[144. Omranipour R, Karbakhsh M, Behforouz A, Neishaboury M, Mahmoodzadeh H, Koma KB, et al. Performance of the Gail Model for Breast Cancer Risk Assessment in Iranian Women. Arch Breast Cancer. 2015; 27–31. doi:10.19187/abc.20152127-31](https://www.zotero.org/google-docs/?kNRxsh)

[145. Solikhah S, Nurdjannah S. Assessment of the risk of developing breast cancer using the Gail model in Asian females: A systematic review. Heliyon. 2020;6: e03794. doi:10.1016/j.heliyon.2020.e03794](https://www.zotero.org/google-docs/?kNRxsh)

[146. ROSTAMI S, RAFEI A, DAMGHANIAN M, KHAKBAZAN Z, MALEKI F, ZENDEHDEL K. Discriminatory Accuracy of the Gail Model for Breast Cancer Risk Assessment among Iranian Women. Iran J Public Health. 2020;49: 2205–2213. doi:10.18502/ijph.v49i11.4739](https://www.zotero.org/google-docs/?kNRxsh)

[147. Husereau D, Drummond M, Augustovski F, de Bekker-Grob E, Briggs AH, Carswell C, et al. Consolidated Health Economic Evaluation Reporting Standards 2022 (CHEERS 2022) statement: updated reporting guidance for health economic evaluations. BMJ. 2022;376: e067975. doi:10.1136/bmj-2021-067975](https://www.zotero.org/google-docs/?kNRxsh)
