# Supplementary material for: Reducing non-communicable diseases among Palestinian populations in Gaza: A participatory comparative and cost-effectiveness modeling assessment
Source: PLOS Glob Public Health. 2024 May 2;4(5):e0003168. doi: 10.1371/journal.pgph.0003168 (PMC11065248; doi:10.1371/journal.pgph.0003168)
Supplement: Attachment — Submitted filename: Response to reviewers.docx [file pgph.0003168.s004.docx]

| **Editor or reviewer comment** | **Edits made to manuscript** | **Location of edits** |
| --- | --- | --- |
| **Editor** |  |  |
| Inconsistency on what is reported in the methods section and result: confidence interval vs. credible interval. "Uncertainty analyses are reflected in the 95% confidence intervals around the mean results.” | Thank you for catching this error. We have corrected the analysis to refer to ‘credible intervals’ throughout. | Results, sensitivity and uncertainty analyses, p. 23 |
|  |  |  |
| **Reviewer #1** |  |  |
| “I appreciate the opportunity to critically review the manuscript titled "Reducing non-communicable diseases among Palestinian populations in Gaza: a participatory comparative and cost-effectiveness modeling assessment." It is an interesting and highly relevant article that analyzes the health situation of a population with very specific conditions that could have a negative impact on their well-being. While it is true that the study has limitations, appropriately addressed by the authors, I believe the obtained results are highly relevant for public health. I present to the authors a series of minor comments for their consideration.” | Thank you. | - |
| “Comment 1: On page 13, it is stated that more than half of the participants (54%) are women, and almost 7 out of 10 participants identified as refugees. Are these proportions representative of the source population? Please briefly discuss this.“ | We have now added a note indicating that the participant demographics were similar to the 2017 population census (most recent available) in Gaza, but we anticipate will no longer be as representative following the October invasion of Gaza in which large portions of the population were killed, and many more became refugees or internally displaced popuations. | Results, characteristics of the study population subsection, p. 13  Discussion, limitations subsection, p. 25 |
| Comment 2: In Table 2 (page 15), I found it concerning that there are no interventions aimed at improving the psychosocial and mental health of this population. I understand that current priorities (January 2024) may differ, but it is an aspect worth briefly discussing. | We now highlight the limitation labeled in Table 2 that our experts in the Delphi process indicated there were insufficient data to evaluate the prevalence of need in this important domain, and that undoubtedly this domain will be of increasing importance following the recent escalation of conflict in Gaza. | Discussion, limitations subsection, p. 25 |
| Comment 3: The political and health situation of the studied population recently changed for reasons beyond the control of the researchers. It would be interesting to briefly discuss the potential impact of these recent events on the burden of the diseases analyzed in this research | Thank you, we have now added a new paragraph to the Discussion to discuss the implications of these recent events on the burden of disease identified in this research. | Discussion, p. 25 |
|  |  |  |
| **Reviewer #2** |  |  |
| The author concluded that high levels of NCD risk factors among Palestinians in Gaza have estimated that several interventions would be expected to reduce the loss of DALYs within common cost-effectiveness thresholds. So, the author conducted a timely analysis. | Thank you. | - |
| [no further comments from Reviewer 2] |  |  |
|  |  |  |
| **Reviewer #3** |  |  |
| Given the events that have been happening in Gaza for sometime, it is a perfect timing to have an article of this kind. This can be used as a reference for implementing interventions not only in Gaza but also in context that have been/ are being ravaged by political instabilities. For that, I would like to congratulate the authors.  This article is presented using a simple and clear language, and there is a good coherence in the flow of information. This ensures easy understanding for many readers irrespective of their professional backgrounds.  The method section is detailed and clear. The results and the discussion are well aligned to the objectives and methods used. | Thank you. | - |
| [no further comments from Reviewer 3] |  |  |

\###
